# Early-Onset Colorectal Cancer: A New Clinical Profile in Patients Treated at a Referral Hospital in Mexico City

**DOI:** 10.7759/cureus.89555

**Published:** 2025-08-07

**Authors:** Ana J Iberri Jaime, Billy Jiménez Bobadilla, Fabiola Hernandez Trejo, Jesus Omar Soto Llanes, Juan Antonio Gonzalez Leon, Lenyn D Sevilla, Oscar Santes

**Affiliations:** 1 Colorectal Surgery, General Hospital of Mexico Dr. Eduardo Liceaga, Mexico City, MEX; 2 Surgery, National Institute of Medical Sciences and Nutrition Salvador Zubiran, Mexico City, MEX

**Keywords:** colorectal cancer, colorectal neoplasms, early detection of cancer, early-onset colorectal cancer, health services accessibility, healthy young adult, late-onset colorectal cancer, retrospective studies

## Abstract

Introduction

Early-onset colorectal cancer (EOCRC), defined as colorectal cancer diagnosed before the age of 50, has exhibited a sustained increase in incidence globally. In Mexico, this rising trend presents significant clinical and diagnostic challenges, particularly in younger patients who often lack traditional risk factors. This study aimed to describe and compare the epidemiological, clinical, histopathological, and therapeutic characteristics of EOCRC versus late-onset colorectal cancer (LOCRC) in a referral hospital in Mexico City.

Methods

We conducted a retrospective observational cohort study at the Hospital General de México, including all patients with histologically confirmed colorectal cancer diagnosed between January 2020 and December 2024. Patients were stratified into two groups based on age at diagnosis: <50 years (EOCRC) and ≥50 years (LOCRC). Variables analyzed included sex, tumor grade, Tumor, Node, Metastasis (TNM) stage, histological subtype, and treatments received (chemotherapy and radiotherapy). Categorical variables were compared using Pearson’s Chi-squared or Fisher’s exact test, while continuous variables were analyzed using the Wilcoxon rank-sum test. A p-value <0.05 was considered statistically significant, and test statistics were reported accordingly.

Results

A total of 583 patients were analyzed: 205 (35.2%) in the EOCRC group and 378 (64.8%) in the LOCRC group. The median age at diagnosis was 42.0 years (IQR: 35.0-46.0) for EOCRC and 61.0 years (IQR: 53.0-68.0) for LOCRC (W=0, p<0.001). No significant differences were observed in sex distribution or histological subtype, with adenocarcinoma being the most common in both groups (>90%). Patients with EOCRC were more likely to present with poorly differentiated tumors (24% vs. 15%, χ²=7.85, p=0.020) and were more frequently diagnosed at advanced stages (stage III or IV: 70%). Paradoxically, patients with EOCRC showed a relatively higher proportion of early-stage disease (30% vs. 19%, χ²=23.32, p=0.003) compared to those with LOCRC. Regarding treatment, patients with EOCRC received more adjuvant chemotherapy (69% vs. 59%, χ²=4.20, p=0.040), while those with LOCRC had higher rates of adjuvant radiotherapy (25% vs. 1.5%, χ²=50.88, p<0.001).

Conclusion

EOCRC represents a distinct clinical and pathological entity compared to LOCRC, characterized by younger age at onset, higher rates of poorly differentiated tumors, and more frequent diagnosis at advanced stages. These findings highlight the need for increased clinical vigilance, age-adjusted screening strategies, and further molecular studies to better understand the underlying biology of EOCRC in younger populations.

## Introduction

Colorectal cancer (CRC) represents a global public health concern, ranking as the third most commonly diagnosed malignancy [1] and the second leading cause of cancer-related deaths worldwide [2]. In Mexico, the situation is particularly worrisome; 2022 data indicate that CRC was the third most common cancer in terms of incidence, with over 16,000 new cases, and the leading cause of cancer mortality, with more than 8,200 deaths [3]. This high rate of lethality, compared to high-income countries, suggests possible gaps in early detection and access to treatment [4].

While CRC incidence in adults over the age of 50 has declined in many nations, an alarming and sustained increase has been documented among individuals under 50 years of age [5], a phenomenon known as early-onset colorectal cancer (EOCRC) [6]. Globally, EOCRC is now estimated to account for 10% to 15% of all CRC diagnoses [1]. Recent projections suggest that within the next decade, one in 10 colon cancers and one in four rectal cancers will be diagnosed in this age group [7].

EOCRC frequently presents with distinct clinicopathological characteristics, including a higher proportion of tumors located in the distal colon and rectum, more aggressive histological features, and diagnosis at more advanced stages [7], which complicates management and worsens prognosis [6]. Despite growing international evidence, data characterizing this disease in the Mexican population remain scarce. Therefore, the aim of this study is to describe and compare the epidemiological, clinical, histopathological, and therapeutic characteristics of patients with EOCRC versus those with late-onset colorectal cancer (LOCRC) in a cohort from a national referral center in Mexico City.

## Materials and methods

Study design

A retrospective observational cohort study was conducted, following the recommendations of the STrengthening the Reporting of OBservational studies in Epidemiology (STROBE) guidelines for observational research. The analysis included patients with a histopathologically confirmed diagnosis of CRC, treated between January 2020 and December 2024 at the Hospital General de México, Mexico City, Mexico. 

Population and sample size

All consecutive patients with a confirmed diagnosis of CRC and a complete medical record during the study period were included. Patients with missing essential clinical or pathological data were excluded. As this was a retrospective census-based design, no a priori sample size calculation was performed. The final cohort consisted of 583 patients.

Variables and data collection

Clinical, histopathological, and therapeutic data were extracted from the institutional electronic medical records. Data coding and cleaning were performed independently by two investigators, with discrepancies resolved by consensus, following a standardized extraction protocol. Patients were classified into two groups based on age at diagnosis: EOCRC (<50 years) and LOCRC (≥50 years). The variables analyzed included: age, sex, histologic subtype, tumor differentiation grade, grouped clinical Tumor, Node, Metastasis (TNM) stage (early, locally advanced, or metastatic), surgical margins, perineural invasion, lymphovascular invasion (LVI), and treatment received (neoadjuvant or adjuvant chemotherapy and radiotherapy). Histologic subtypes with fewer than five cases were grouped under “Other.” The clinical TNM stage was recategorized as follows: early stage (0, I, IIA, IIB), locally advanced (IIIA, IIIB, IIIC), and metastatic (IV).

Statistical analysis

Statistical analysis was performed using R software (version 4.4.1, R Foundation for Statistical Computing, Vienna, Austria, https://www.R-project.org/). Categorical variables were described using absolute frequencies and percentages; continuous variables were expressed as medians and interquartile ranges (IQR). Group comparisons were made using the Wilcoxon rank-sum test (age) and the Pearson Chi-square test or Fisher’s exact test for categorical variables, as appropriate. P-values and test statistics (W or χ²) were reported. A p-value <0.05 was considered statistically significant.

Ethical considerations

The study was classified as minimal risk. Confidentiality was maintained through data anonymization. Individual informed consent was not required. The principles of the Declaration of Helsinki were followed. Variables with missing data were analyzed using available case analysis, without data imputation.

## Results

**Figure 1:**
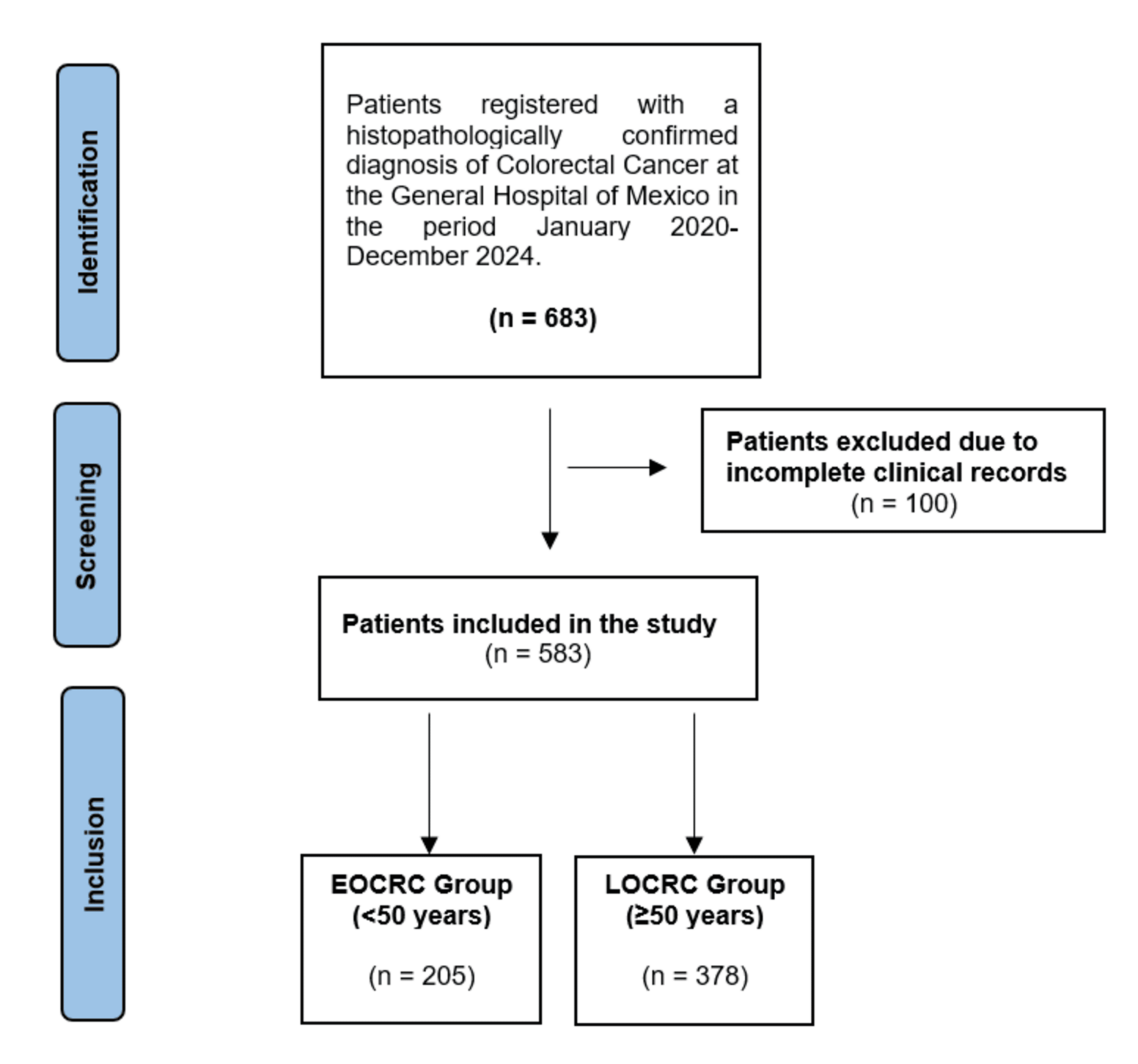
Flow diagram of patient selection included in the institutional cohort with confirmed diagnosis of colorectal cancer between 2020 and 2024. The diagram shows classification into groups based on age at diagnosis (<50 years vs. ≥50 years).

A total of 583 patients with a confirmed diagnosis of CRC were included. Of these, 205 (35.2%) were classified in the EOCRC group (<50 years), and 378 (64.8%) in the LOCRC group (≥50 years) (Figure 1).

**Figure 2:**
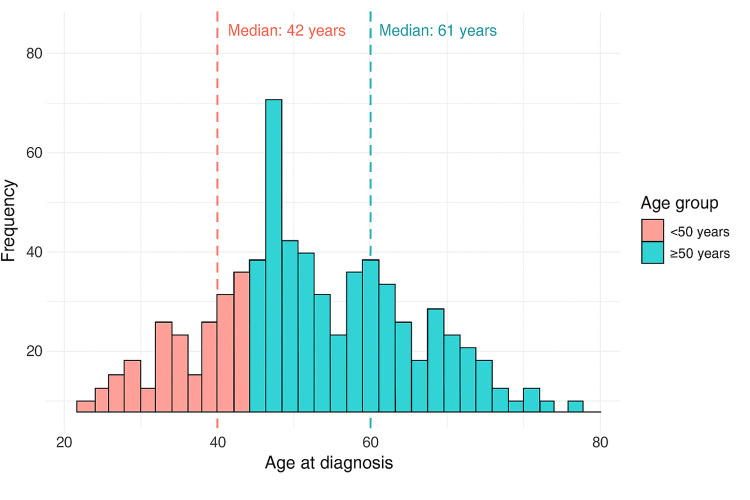
Age distribution at diagnosis of colorectal cancer, stratified by age group

As shown in Figure 2, the median age was 42.0 years (IQR: 35.0-46.0) for patients with EOCRC and 61.0 years (IQR: 53.0-68.0) for patients with LOCRC, with a statistically significant difference (p<0.001; W=0).

No significant differences were observed in sex distribution (50% female patients in EOCRC vs. 48% in LOCRC; p=0.854; χ²=0.03).

Adenocarcinoma was the predominant histological type in both groups (91% vs. 90%; p=0.387; Fisher’s exact test). Stratified descriptive analysis by tumor location showed a higher frequency of rectal cancer in EOCRC (42%) compared to LOCRC (34%).

Clinical stage at diagnosis showed significant differences: 70% of patients with EOCRC presented with advanced disease (35% stage III and 35% stage IV), while this proportion was slightly higher in the LOCRC group (81% in stages III-IV). However, the EOCRC group had a greater proportion of early-stage disease (30% vs. 19%; p=0.003; χ²=23.32).

Regarding tumor differentiation grade, poorly differentiated tumors were more frequent in the EOCRC compared to the LOCRC group (24% vs. 15%; p=0.020; χ²=7.85). No differences were found in the surgical margin status (p=0.241; χ² = 1.37) or perineural invasion (p=0.205; χ²=1.61). LVI was more common in the LOCRC than the EOCRC group (59% vs. 45%; p=0.005; χ²=8.04).

In terms of treatment, patients with LOCRC received neoadjuvant chemotherapy (58% vs. 42%; p<0.001; χ²=13.54) and adjuvant radiotherapy (25% vs. 1.5%; p<0.001; χ²=50.88) more frequently than those with EOCRC. In contrast, adjuvant chemotherapy was more commonly administered in EOCRC (69% vs. 59%; p = 0.040; χ² = 4.20). No significant differences between the two groups were observed in the use of neoadjuvant radiotherapy (p=0.600; χ²=0.27). These findings are summarized in Table 1.

**Table 1:** Summary of demographic, clinical, pathological, and treatment characteristics of patients with colorectal cancer, stratified by age group (<50 vs. ≥50 years) ^1^Median (Q1, Q3); n (%); ^2^Wilcoxon rank sum test; ^3^Pearson’s Chi-squared test; ^4^Fisher’s exact test; ^5^Chi-squared test with simulated p-value The table compares demographic, histopathological, and therapeutic characteristics between patients younger than 50 years (n=205) and those aged 50 years or older (n=378) diagnosed with gastrointestinal tumors. Continuous variables are expressed as median (Q1, Q3) and were compared using the Wilcoxon rank-sum test. Categorical variables are expressed as frequency and percentage, and were analyzed using Pearson’s Chi-squared test, Chi-squared test with simulated p-value, or Fisher’s exact test, as appropriate. The corresponding p-values for each comparison are reported.

Characterístics	<50 years (N=205)	≥50 Years (N=378)	p-value
Age (years)	42.0 (35.0, 46.0)^1^	61.0 (53.0, 68.0)^1^	<0.001^2^
Sex			0.854^3^
Female	103 (50%)	181 (48%)	
Male	102 (50%)	197 (52%)	
Histopatology			0.387^4^
Adenocarcinoma	186 (91%)	341 (90%)	
Mucinous adenocarcinoma	14 (6.8%)	20 (5.3%)	
Gastrointestinal Stromal Tumor (GIST)	3 (1.5%)	2 (0.5%)	
Others	2 (1.0%)	15 (4.0%)	
Tumor, Node, Metastasis (TNM) Stage			0.003^5^
0, I, IIA, IIB	62 (30%)	72 (19%)	
IIIA, IIIB, IIIC	72 (35%)	146 (39%)	
IV	71 (35%)	160 (42%)	
Tumor grade			0.020^3^
Poorly differentiated	48 (24%)	57 (15%)	
Moderately differentiated	131 (65%)	259 (69%)	
Well differentiated	22 (11%)	62 (16%)	
Surgical margins			0.241^3^
Negative	96 (61%)	149 (56%)	
Positive	61 (39%)	119 (44%)	
Perineural invasion			0.205^3^
Absent	133 (84%)	223 (79%)	
Present	25 (16%)	59 (21%)	
Lymphovascular invasion (LVI)			0.005^3^
Absent	87 (55%)	123 (41%)	
Present	71 (45%)	177 (59%)	
Neoadjuvant chemotherapy			<0.001^3^
Not given	118 (58%)	158 (42%)	
Given	86 (42%)	217 (58%)	
Neoadjuvant radiotherapy			0.6^3^
Not given	122 (60%)	214 (57%)	
Given	82 (40%)	160 (43%)	
Adjuvant chemotherapy			0.04^3^
Not given	64 (31%)	155 (41%)	
Given	141 (69%)	220 (59%)	
Adjuvant radiotherapy			<0.001^3^
Not given	201 (99%)	283 (75%)	
Given	3 (1.5%)	92 (25%)	

## Discussion

This study confirms that EOCRC is a clinically relevant entity in the Mexican population treated at a referral center, with findings that both align with and challenge those reported in the international literature.

The most striking finding is the 35.2% prevalence of EOCRC in our cohort, which is markedly higher than the 10-15% reported in population-based registries from the US [1] and Europe [5]. This elevated proportion is most likely explained by referral center bias, as tertiary hospitals like ours tend to concentrate on more complex, atypical, or early-onset cases referred from lower levels of care. Nonetheless, this figure highlights the burden that EOCRC represents for Mexico’s highly specialized healthcare system.

Our results confirm that EOCRC is predominantly diagnosed at advanced stages (70% in stage III/IV), a pattern consistent with global literature attributing this trend to low clinical suspicion and diagnostic delays in younger patients [6]. However, our study reveals an interesting duality: the EOCRC group also had a significantly higher proportion of early-stage diagnoses (30% vs. 19%). This may suggest a heterogeneous clinical presentation, where a subset of young patients with alarming symptoms (e.g., profuse rectal bleeding) undergoes more aggressive and prompt workup than older adults, leading to earlier detection.

The observed histopathological profile with a higher frequency of poorly differentiated tumors in the EOCRC group aligns with descriptions of EOCRC as a biologically more aggressive entity [7]. However, the finding of less frequent LVI in the same group is counterintuitive and contradicts previous reports associating EOCRC with more adverse prognostic features [7]. This discrepancy may be due to the higher proportion of early-stage cases in our EOCRC cohort, potentially diluting the overall prevalence of LVI. This finding warrants further investigation, including pathology report validation and molecular analysis. The differences in adjuvant radiotherapy, despite a higher frequency of rectal tumors in the EOCRC group, suggest complex treatment decisions, possibly influenced by factors like neoadjuvant approaches or patient-specific considerations not captured in this analysis.

Finally, the findings of this study carry important public health implications in Mexico. The current national Clinical Practice Guideline, published in 2008, recommends starting CRC screening at age 50 for average-risk individuals [8,9]. This recommendation is outdated given the growing evidence on EOCRC and stands in contrast to the American Cancer Society (ACS) guidelines, which have recommended initiating screening at age 45 since 2018 [10]. The high burden of advanced disease observed in our cohort of young patients provides strong local evidence supporting the urgent need to review and update CRC screening policies in Mexico.

Limitations of this study include its retrospective, single-center nature, and the referral bias already discussed. Additionally, we did not collect data on modifiable risk factors such as physical inactivity [11] and obesity [12], nor on the molecular profiles of tumors. Literature has shown that these profiles differ in EOCRC [8] and include specific alterations such as tumor protein p53 (TP53) gene loss [13,14], which are key to disease pathogenesis but were not explored in this study.

## Conclusions

EOCRC in this Mexican cohort is characterized by frequent presentation at advanced stages and with more aggressive histological features. These findings underscore the need for a high index of clinical suspicion in young patients with persistent gastrointestinal symptoms. Despite its limitations, the data presented serve as a call to action for Mexican health authorities to re-evaluate current clinical practice guidelines. Specifically, they highlight the need to consider lowering the starting age for CRC screening in order to align national policies with current scientific evidence and help mitigate the impact of this emerging disease.

## References

[1] Eng C, Yoshino T, Ruíz-García E (2024). Colorectal cancer. Lancet.

[2] Siegel RL, Miller KD, Wagle NS, Jemal A (2023). Cancer statistics, 2023. CA Cancer J Clin.

[3] Bray F, Laversanne M, Sung H, Ferlay J, Siegel RL, Soerjomataram I, Jemal A (2024). Global cancer statistics 2022: GLOBOCAN estimates of incidence and mortality worldwide for 36 cancers in 185 countries. CA Cancer J Clin.

[4] Pan American Health Organization (2025). Colorectal Cancer Screening in Latin America and the Caribbean. https://iris.paho.org/bitstream/handle/10665.2/28552/PAHONMH16003-eng.pdf?sequence=1&isAllowed=y.

[5] Patel SG, Karlitz JJ, Yen T, Lieu CH, Boland CR (2022). The rising tide of early-onset colorectal cancer: a comprehensive review of epidemiology, clinical features, biology, risk factors, prevention, and early detection. Lancet Gastroenterol Hepatol.

[6] Sinicrope FA (2022). Increasing incidence of early-onset colorectal cancer. N Engl J Med.

[7] Zaborowski AM, Abdile A, Adamina M (2021). Characteristics of early-onset vs late-onset colorectal cancer: a review. JAMA Surg.

[8] Akimoto N, Ugai T, Zhong R (2021). Rising incidence of early-onset colorectal cancer - a call to action. Nat Rev Clin Oncol.

[9] Mexican Social Security Institute (2009). Clinical Practice Guideline: Early Detection and Diagnosis of Non-Hereditary Colon and Rectal Cancer in Adults at the Primary, Secondary, and Tertiary Levels of Care. Clinical Practice Guide, Early Detection and Diagnosis of Non-Hereditary Colon and Rectal Cancer in Adults in Primary, Secondary, and Third Levels of Care, Mexico (Report in Spanish).

[10] Wolf AM, Fontham ET, Church TR (2018). Colorectal cancer screening for average-risk adults: 2018 guideline update from the American Cancer Society. CA Cancer J Clin.

[11] Nguyen LH, Liu PH, Zheng X (2018). Sedentary behaviors, TV viewing time, and risk of young-onset colorectal cancer. JNCI Cancer Spectr.

[12] Liu PH, Wu K, Ng K (2019). Association of obesity with risk of early-onset colorectal cancer among women. JAMA Oncol.

[13] Willauer AN, Liu Y, Pereira AA (2019). Clinical and molecular characterization of early-onset colorectal cancer. Cancer.

[14] Kim JE, Choi J, Sung CO (2021). High prevalence of TP53 loss and whole-genome doubling in early-onset colorectal cancer. Exp Mol Med.

